# In vitro mixing formulas for antimicrobial calcium sulfate beads for point-of-care applications: an additional update to formulas

**DOI:** 10.5194/jbji-10-309-2025

**Published:** 2025-08-15

**Authors:** Edward J. McPherson, Andrew J. Wassef, Matthew V. Dipane, Madhav Chowdhry

**Affiliations:** 1 Department of Orthopaedic Surgery, David Geffen School of Medicine, UCLA, Los Angeles, CA 90404, USA; 2 Long Beach Lakewood Orthopedic Institute, Long Beach, CA 90808, USA; 3 Department of Orthopaedic Surgery, Jawaharlal Nehru Medical College, AMU, Aligarh 202001, India; 4 Nuffield Department of Continuing Education, University of Oxford, Oxford, OX1 2JA, United Kingdom

## Abstract

This communication updates a previous report describing mixing formulas for antimicrobial agents into a synthetic, dissolvable calcium sulfate (CaSO_4_) product to create small-sized beads (3–6 mm). Antimicrobial-loaded calcium sulfate (ALCS) beads are applied as surgeon-directed, point-of-care use in cases of periprosthetic joint infection (PJI), fracture-related infection (FRI), and other related bone site infections. This treatment technique has been utilized more frequently in the last decade to combat microbial musculoskeletal infections (MSIs). With the widening spectrum of identified microbes encountered in MSIs, we have provided additional antimicrobial formulas to be used singularly and in combination using our in vitro mixing lab protocol previously published. This communication reports 20 additional agents/combinations tested using our protocol to arrive at a standard mix formula or, when necessary, a modified mixing method. Most mixing formulas derived were for antimicrobials effective against fungi and mycobacterium species.

## 1 Introduction

Musculoskeletal infections (MSIs) are increasingly challenging to treat. The reasons for this include the following: the many ongoing adaptive resistance mechanisms selected out by the persistent use of systemic, broad-spectrum antimicrobial agents, the increasing use of immune modulating agents deployed in the treatment of immune-related disorders, and the insertion of sophisticated metallic/ceramic implants for musculoskeletal reconstruction that can harbor micro-organisms (Kurtz et al., 2008; Hamad et al., 2022; Gallo et al., 2014). Treatment has required innovation. In the specific areas of periprosthetic joint infection (PJI), fracture-related infection (FRI), and other bone infection sites, one tactic used for treatment involves local delivery of antimicrobials via dissolvable antimicrobial-loaded calcium sulfate (CaSO_4_) in a small bead form (3–6 mm). The beads are made by the surgeon at the time of surgery, with point-of-care delivery to the infected site (McPherson et al., 2013; Abosala and Ali, 2020; Howlin et al., 2015).

In a previous article, we described mixing various antimicrobial agents into a synthetic, commercially pure CaSO_4_ product in a laboratory setting (McPherson et al., 2021; McPherson et al., 2022). In cursory review, this concept seems simple, but in reality, the process is tedious. The reported mixing formulas were developed to provide surgeons with access to the many available options for treatment. Because of a widening spectrum of identified microbes encountered in MSIs, we study additional agents singularly and in combination using our in vitro mixing lab protocol which simulates the operating room environment (Chowdhry et al., 2024; Nafea et al., 2024). This report provides 20 additional mixing formulas. We report antimicrobial doses used in our standardized mixing technique. Further, when encountering difficulty in mixing a specific agent(s), we developed a modified mixing technique to create the loaded bead.

## 2 Methods

**Figure 1:**
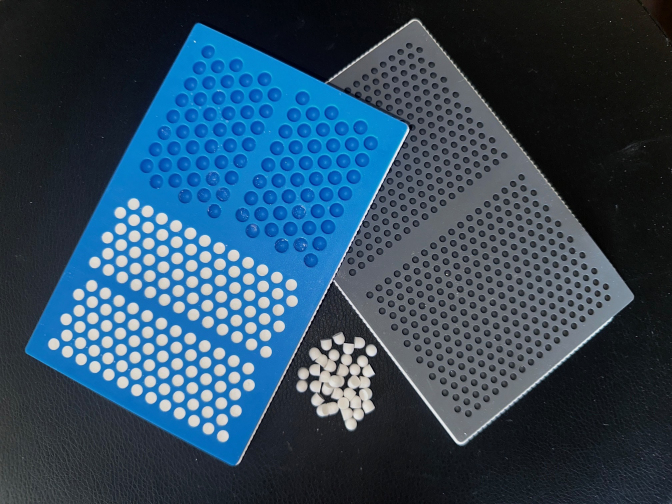
Photograph of CaSO_4_ antimicrobial bead mat. The antimicrobial-loaded CaSO_4_ paste is placed into the bead mat, creating either 3.0, 4.8, or 6.0 mm beads. For study, the paste was spread into the 4.8 mm hemisphere molds. The bead mat was left on the countertop to set, as it would in the operating room. Time measurements were recorded to establish a set time for each formulation.

The study technique employed has been previously described in detail (McPherson et al., 2021). For each agent or combination evaluated, we performed the test a minimum of 3 times. The mix time and set time (defined below) for each agent/combination was averaged for the reported result. The CaSO_4_ product used for study was Synthecure^®^ (Austin Medical Ventures, Memphis, TN), a pharmaceutically derived product. We used the 10 cm^3^ bead kit that consisted of CaSO_4_ hemihydrate powder that is mixed with 5 cm^3^ of sterile saline. When mixed, the unloaded CaSO_4_ product makes a paste volume of 10 cm^3^. The mixed product is spread into a sterile silicone bead mat, allowing for the fabrication of 3, 4.8, or 6 mm beads. For our study, we selected the 4.8 mm bead size. The bead mat and bead product formed are shown in Fig. 1.

In the majority of antimicrobial formulae, the sterile powdered form of the agent was pre-mixed into the CaSO_4_ powder, after which the sterile saline was added. Mixing was performed in the 6.5 cm diameter mixing bowl with a stir stick (components provided in the kit) until a paste consistency devoid of clumps was achieved. The paste product was transferred via the spatula to the bead mat. We measured the time from initial mix until the product became consistently smooth for matting (defined as mix time). Once spread into the bead mat, the product was left to set on the laboratory table in standard conditions of 21.1 °C (70 °Fahrenheit) and 40 % humidity. We defined the product as “set” when we could bend the bead mat as it was turned upside down and the beads would fall onto the table. A second confirmatory test included a digital bead compression test when the bead was on the table. When “fully set”, the bead would not collapse by compressing the bead with the tip of the index finger. The measured “set time” was defined as the time from initial mix until the bead dropped out of the bead mat and was confirmed as fully set via the digital compression test.

Exceptions to the powder mix method noted above were liquid tobramycin and gentamicin. In many centers, both tobramycin and gentamicin come conveniently pre-mixed in 2 mL vials and can be used as a substitute to the sterile saline that is included in the kit. Liquid tobramycin (or gentamicin) provides an advantage in that other antimicrobial powders can be added to the CaSO_4_ powder, allowing combination formulas to be created.

During the standardized mix tests, we found difficulty in mixing numerous antimicrobial agents. In these cases, we encountered long set times, ranging from 30 min to over 3 h. We also experienced antimicrobial/CaSO_4_ combinations that set too quickly to fabricate a quality bead product. For these irregular set times, we experimented with alternative mixing methods with minimal dose alterations. We arrived at modified mixing techniques to provide reasonable set times, for all of these antimicrobial agents. The modified mixing instructions are described for each specific mix, with individualized mixing notes. The modified mixing instructions are grouped into the category of “modified-mix protocols”.

## 3 Results

The standard mix times creating antimicrobial-loaded calcium sulfate (ALCS) beads are reported in Table 1. The two measured times include the mix time and set time. In the cases where liquid agents were substituted for saline, we also report mix time and set time in a similar fashion. For the standard mix method, all tested antimicrobial(s) were set within 4 to 13 min. The recorded mix times and set times tested between testing authors varied no more than 60 s in all agents tested.

**Table 1:** Synthecure set times with antimicrobial/combinations – standard mix technique.

Antibiotic(s)/ antifungal	Dose (mg)	Powder (P)/ liquid (L)	Avg mix time (min:s)	Avg set time (min:s)	Notes
Control (no antimicrobials)	n/a	n/a	0:30	4:10	
Anidulafungin	100	P	0:35	6:00	Easy to mix. Smooth paste. Mat by 3 min.
Anidulafungin/ tobramycin/ vancomycin	100/240/1000	P/L/P	0:40	12:30	Tobramycin is 80 mg per 2 mL vial. Add 6 mL tobramycin liquid to powder. Mixes well, runny. Wait 3 min to mat.
Caspofungin	50	P	0:35	4:30	Smooth paste, easy to mix. Need to mat by 2:30 as it gets harder to spread.
Caspofungin/ tobramycin/ vancomycin	50/240/1000	P/L/P	0:48	9:53	Tobramycin is 80 mg per 2 mL vial. Add 6 mL tobramycin liquid to powder. Slightly runny. Wait until 2 min to mat. Fully mat by 4:30.
Micafungin	50	P	0:35	5:15	Smooth, easy to mix paste. Finish matting by 3:15.
Micafungin/ tobramycin/ vancomycin	50/240/1000	P/L/P	0:45	10:35	Tobramycin is 80 mg per 2 mL vial. Add 6 mL tobramycin liquid to powder. Slightly runny mix. Firms up and spreads easily at 2:30. Finish matting by 4:30.

n/a – not applicable.

The modified mix protocols and described methods are reported in Table 2. For each agent/combination, we describe the modified mixing technique. The mix times and set times using the modified mix protocols are recorded. The recorded mix times and set times between the two testing authors varied no more than 60 s in all agents tested.

**Table 2:** Synthecure set times with antimicrobial/combinations – modified mix protocols.

Antibiotic(s)/ antifungal	Dose (mg)	Powder (P)/ liquid (L)	Avg mix time (min:s)	Avg set time (min:s)	Notes
Amikacin/ vancomycin	500/1000	L/P	0:30	27:00	Amikacin is 500 mg per 2 mL vial. Add 2 mL amikacin and an additional (exactly) 2.5 mL of saline to powder and mix. Makes a dough-like paste. Must finish matting by 2:30.
Ethambutol	1200	P	0:42	15:20	Take 400 mg tablets × 3 and pestle. Add to CaSO_4_ powder. Smooth mix, easy to spread paste. Can mat immediately.
Ethambutol/ tobramycin/ vancomycin	1200/240/1000	P/L/P	0:48	18:00	Take 400 mg tablets × 3 and pestle. Add to CaSO_4_ powder. Tobramycin is 80 mg per 2 mL vial. Add 6 mL tobramycin liquid to powder. Runny mix, wait 3–4 min to mat, spreads easily at that point.
Isoniazid	300	P	0:30	4:20	Take 300 mg tablet × 1 and pestle. Add to CaSO_4_ powder. Mix quickly. Paste is dough-like. Mat at 30 s. Finish matting by 2 min.
Isoniazid/ tobramycin/ vancomycin	300/240/1000	P/L/P	0:40	10:20	Take 300 mg tablet × 1 and pestle. Add to powder. Tobramycin is 80 mg per 2 mL vial. Add 6 mL tobramycin liquid to powder. Slightly runny mix. Mat at 2 min. Finish matting by 4 min.
Itraconazole	200	P	0:30	5:00	Open one itraconazole capsule and add to CaSO_4_ powder. Paste is gritty but easy to spread. Mat at 30 s. Finish matting by 2:30.
Itraconazole/ tobramycin/ vancomycin	200/240/1000	P/L/P	0:40	8:45	Open one itraconazole capsule and add to powder. Tobramycin is 80 mg per 2 mL vial. Add 6 mL tobramycin liquid to powder. Slightly runny but easy to mix and spread. Mat at 2 min. Finish by 4 min.
Meropenem/ gentamicin	1000/240	P/L	0:30	20:00	Gentamicin is 80 mg per 2 mL vial. Add 6 mL of gentamycin liquid to CaSO_4_ powder and mix for 30 s. Wait 4 min and then add 1 g of meropenem powder. Mix for an additional 30 s before matting.
Posaconazole	100	L	0:30	15:15	Posaconazole comes in 18 mg mL^−1^ liquid. Use 5.5 mL and add to CaSO_4_ powder. Smooth, somewhat tacky mix. Can mat immediately after mixing. Mat by 4 min.
Posaconazole/ vancomycin	100/1000	L/P	0:57	14:50	Posaconazole comes in 18 mg mL^−1^ liquid. Use 5.5 mL and add to powder. Must mix quickly and vigorously to produce a smooth paste. Finish matting by 3:15.

**Table 2:** Continued.

Antibiotic(s)/ antifungal	Dose (mg)	Powder (P)/ liquid (L)	Avg mix time (min:s)	Avg set time (min:s)	Notes
Tobramycin/ vancomycin/ ketorolac (Sekar et al., 2023)	600/1000/60	P/P/L	0:45	21:00	Ketorolac comes in 30 mg mL^−1^ liquid. Add 2 mL ketorolac plus 3 mL of additional saline to powder. Slightly loose mix. Wait 2 min to mat. Finish matting by 5 min.
Tobramycin/ vancomycin/ ketorolac (Sekar et al., 2023) *Alternative mix*	600/1000/60	P/P/L	1:00	14:30	Add 2 mL ketorolac plus 3 mL of additional saline to vancomycin/CaSO_4_ powder and mix for 30 s. Wait 30 s and then add 600 mg tobramycin powder. Slightly loose mix. Wait 2 min to mat. Finish matting by 5 min.
Voriconazole/ vancomycin/ amphotericin B/ tobramycin	200/1000/250/240	P/P/P/L	1:00	19:30	Tobramycin is 80 mg per 2 mL vial. First add 6 mL tobramycin liquid into 200 mg voriconazole powder bottle and dissolve (10 min). Once fully dissolved, use that liquid as mixing solution. Runny mix. Wait 2:30 to mat. Finish matting by 4:30.
Voriconazole/ vancomycin/ amphotericin B/ tobramycin	200/1000/250/320	P/P/P/L	1:00	29:45	Tobramycin is 80 mg per 2 mL vial. First add 8 mL tobramycin liquid into 200 mg voriconazole powder bottle and dissolve (10 min). Once fully dissolved, use that liquid as mixing solution. Runny mix. Wait 2:00 to mat. Finish matting by 4:30.

## 4 Discussion

This supplemental compendium of mixing formulas is by no means a recommended treatment algorithm for MSIs. However, use of local antimicrobial delivery via ALCS beads is described globally (Tang et al., 2022; Kallala et al., 2018; Sahoo et al., 2024; Ferrando et al., 2017; Whitlark et al., 2016; Healy et al., 2012). Further, there are recent concerns by musculoskeletal infection experts that overuse of systemic antimicrobial agents adversely affects the human microbiome, creating concerning downstream consequences to the human host (Patangia et al., 2022; Aboushaala et al., 2023; Ribeiro et al., 2020). Local antimicrobial delivery may become a focus area for future MSI treatment. Studies are slowly emerging that show local delivery via ALCS beads may reduce the duration of parenteral antimicrobial therapy (McPherson et al., 2013). Until recently, antibiotic and antifungal formulas used to make ALCS beads have been within the purview of surgeon-to-surgeon or surgeon-to-company representative sidebar discussions. We bring these discussions to the forefront and include these additional formulas for ongoing academic debate. Moreover, we want to educate physicians treating bone and joint infections that these antimicrobial formulas exist. Physicians uninformed with this technique of antimicrobial delivery may potentially over-treat with adjuvant parenteral antimicrobials, causing untoward patient side effects. The description of these mixing formulas serves as a starting basis for discussion, providing a reference point for set times.

Since we first reported upon mixing formulas for antimicrobial-loaded CaSO_4_ beads, multiple CaSO_4_ bead products have emerged for clinical application. We highlight that every CaSO_4_ product sold for medical use as a bead product is unique and that the mixing formulas we have reported previously and here within are specific to the Synthecure product. Although generally applicable, our mixing data cannot be uniformly translated to other products. For mined and refined products, impurities (including heavy metal elements) impart variations in set times and also limit the mixing of many antimicrobials/combinations (Mu et al., 2019; Fernandes et al., 2021). For synthetic products, the proprietary formulas created provide efficient CaSO_4_ setting with antimicrobial agents and are unique to each product. The chemical reactions affecting these differences are not exactly known, but, empirically, product development has focused on efficient antimicrobial loading and elution. Each product is proprietary with unique performance (McPherson et al., 2022). We acknowledge that there are various pharmaceutical suppliers of antimicrobial agents, and mixing set times may vary slightly based upon added excipients. This study does not report upon elution of these agents from mixed beads and will require further testing. In a simulated in vitro synovial large joint model, we have documented high effective antimicrobial levels of vancomycin and tobramycin that are sustained for weeks (McPherson et al., 2022). However, for this study, we have not yet measured the effective antimicrobial levels of these reported agents in this model. This will be a topic of future study. We also warn that successful mixing and setting of antimicrobial agents is impaired when using hybrid bead products that contain CaSO_4_ in combinations with hydroxyapatite and/or β -tricalcium phosphate. In such products, the chemical interactions become complex, and from anecdotal experience, these additional agents distinctly impair mixing, setting, elution, and bead resorption. Finally, it is unlikely that novel carrier agents will enter the market in the near future due to the inherent challenges globally to approve a delivery agent. Thus, we advocate focused study and scrutiny of calcium sulfate for local antimicrobial delivery to musculoskeletal infection sites.

It remains our future goal to establish an open-access site such that all physicians and surgeons worldwide can report upon mixing formulas to educate and assist other physicians with difficult infection cases. Additionally, we hope to one day see the expansion of mixing formulas that extend beyond the current realm of antibiotic and antifungal chemicals and transition to non-specific antimicrobial agents.

## 5 Summary

We performed a mixing lab study describing 20 additional mixing formulas with mixing/set times in creating antimicrobial CaSO_4_ beads used for local delivery in periprosthetic joint infection, fracture-related infections, and other bone site infections. Most new formulas were focused on antifungal and antimycobacterial therapy. This information is to be utilized as an open-access reference for all physicians involved in the treatment of musculoskeletal infection.

## Data Availability

All data in this study can be found in Tables 1 and 2.
